# *In vitro* evaluation of the osteogenic and antimicrobial potential of porous wollastonite scaffolds impregnated with ethanolic extracts of propolis

**DOI:** 10.3389/fbioe.2024.1321466

**Published:** 2024-02-01

**Authors:** Ana Isabel Moreno Florez, Sarita Malagon, Sebastian Ocampo, Sara Leal-Marin, Edgar Alexander Ossa, Birgit Glasmacher, Claudia Garcia, Alejandro Pelaez-Vargas

**Affiliations:** ^1^ Grupo de Materiales Cerámicos y Vítreos, Universidad Nacional de Colombia Sede Medellín, Medellín, Colombia; ^2^ Grupo GIOM, Facultad de Odontología, Universidad Cooperativa de Colombia, Sede Medellín, Colombia; ^3^ Institute for Multiphase Processes (IMP), Leibniz University Hannover, Garbsen, Germany; ^4^ Lower Saxony Center for Biomedical Engineering, Implant Research and Development, Hannover, Germany; ^5^ School of Applied Sciences and Engineering, Universidad Eafit, Medellín, Colombia

**Keywords:** bone tissue engineering, 3D printing, human bone marrow stem cells, antimicrobial activity, wollastonite

## Abstract

**Context:** The development of porous devices using materials modified with various natural agents has become a priority for bone healing processes in the oral and maxillofacial field. There must be a balance between the proliferation of eukaryotic and the inhibition of prokaryotic cells to achieve proper bone health. Infections might inhibit the formation of new alveolar bone during bone graft augmentation.

**Objective:** This study aimed to evaluate the *in vitro* osteogenic behavior of human bone marrow stem cells and assess the antimicrobial response to 3D-printed porous scaffolds using propolis-modified wollastonite.

**Methodology:** A fractional factorial design of experiments was used to obtain a 3D printing paste for developing scaffolds with a triply periodic minimal surface (TPMS) gyroid geometry based on wollastonite and modified with an ethanolic propolis extract. The antioxidant activity of the extracts was characterized using free radical scavenging methods (DPPH and ABTS). Cell proliferation and osteogenic potential using Human Bone Marrow Stem Cells (bmMSCs) were assessed at different culture time points up to 28 days. MIC and inhibition zones were studied from single strain cultures, and biofilm formation was evaluated on the scaffolds under co-culture conditions. The mechanical strength of the scaffolds was evaluated.

**Results:** Through statistical design of experiments, a paste suitable for printing scaffolds with the desired geometry was obtained. Propolis extracts modifying the TPMS gyroid scaffolds showed favorable cell proliferation and metabolic activity with osteogenic potential after 21 days. Additionally, propolis exhibited antioxidant activity, which may be related to the antimicrobial effectiveness of the scaffolds against *S. aureus* and *S. epidermidis* cultures. The mechanical properties of the scaffolds were not affected by propolis impregnation.

**Conclusion:** These results demonstrate that propolis-impregnated porous wollastonite scaffolds might have the potential to stimulate bone repair in maxillofacial tissue engineering applications.

## 1 Introduction

Restorative dentistry is well established in the field of dentistry and involves biomaterials, such as metallic alloys, polymers, composites, and ceramics, to restore lost tissue function and aesthetics (Anusavice et al., 2013). After more than a century of biomaterial development, clinicians have found that synthetic materials often fail to satisfy patients’ requirements for both aesthetics and function. This observation has led to an extended definition that incorporates concepts from regenerative dentistry, wherein three key requirements are present: a) synthetic or biological scaffolds, b) progenitor/stem cells, and c) the presence of inductive morphogenic signals (Nakashima and Akamine, 2005). The literature describes two distinct approaches -cellular and acellular-which have rapidly transitioned from laboratory research to clinical applications. This rapid progress observed can be partly attributed to the minimally invasive access and easy observation available in the oral cavity during the healing process (Velasquez-Plata, 2022).

Currently, regenerative dentistry encompasses a range of bone repair applications, from extensive reconstruction due to factors such as trauma or tumors, to more localized issues such as dehiscence and fenestration. One of the most common challenges faced by clinicians is the bone atrophy of the dentoalveolar process resulting from the early loss of teeth, as it affects treatments such as orthodontics, implants, or restorations (Mayer et al., 2016; Bontá et al., 2023). The recovery of volume and height in the dentoalveolar process is influenced by complex interactions involving both hard and soft tissues, acute/subacute/chronic infections, and the immunological response of bone (Albrektsson et al., 2023). These factors are modulated within microenvironments where the metabolism of mesenchymal stem cells (MSCs), balance of bacteria/hosts, and signaling processes all play roles in maintaining homeostasis (Shanbhag et al., 2022).

Regenerative dentistry based on scaffolds is a promising approach for tissue and organ replacement. Adult stem cells, particularly MSCs, are commonly used in conjunction with scaffolds (Zhang and Chen, 2014; El-Gendy et al., 2021). However, the isolation, expansion, and differentiation of these cells pose clinical challenges (Pelaez-Vargas et al., 2012). The multilineage potential was described previously as a hyper-hierarchical lineage that starts with marrow stromal fibroblastic stem cells. Through appropriate stimulation, these cells progress through a developmental sequence of osteoprogenitor, preosteoblast, osteoblast, and osteocyte cells (Pittenger et al., 1999).

The most convenient sources of human MSCs are bone marrow aspirates taken from the iliac crest under local anesthesia or a combination of bone tissue explants and bone marrow obtained from orthopedic corrective hip surgeries (Khorasani et al., 2021). In all cases, informed consent is required to collect and use these biological materials, which would otherwise be discarded. There is also an increasing focus on human dental MSCs, classified based on their niche within the dental environment (Machado et al., 2012). In the context of regenerative dentistry, scaffolds have been extensively studied *in vitro* and *in vivo*, with an emphasis on factors such as scale (micro, nanometric, or hyper-hierarchical), origin (natural or synthetic), product type (massive or customized), manufacturing techniques (including 3D printing and bioprinting) (Nikolova and Chavali, 2019), functionality (3D, 3D functionalized, and 4D) (Soleymani and Naghib, 2023; Wan et al., 2023), geometries (periodic or non-periodic) and composition (polymer, metallic, or ceramic) (Teixeira et al., 2009; Higuita-Castro et al., 2012; Martin et al., 2019; Moreno et al., 2023). However, ceramic-based 3D scaffolds have received relatively less attention. A variety of studies have explored the potential of wollastonite-based scaffolds in bone tissue engineering. Wollastonite, a calcium silicate, possesses properties such as bioactivity, biocompatibility, and antibacterial effects (Shao et al., 2017; Gao et al., 2023; Moreno et al., 2023; Yue et al., 2023). A combination of compositions and complex geometries (TPMS—triply periodic minimal surfaces) might be favorable for cell behavior (Ramírez et al., 2019) as they can closely mimic natural cortical bone tissues, facilitate good nutrient transfer, and promote stress shielding.

Currently, functionalized scaffolds are at the center of attention and have evolved from approaches aimed at creating 3D structures that mimic the extracellular matrix and serve as deposits for calcium, phosphate, silica, and other ions involved in the later stages of bone healing (Teixeira et al., 2009). However, this approach has had limitations that have been gradually addressed by combining strategies, such as synthetic antibiotic substance carriers with a local response that impacts bacterial proliferation through processes such as inhibiting cell wall synthesis, breaking the osmotic barrier of cell membranes, or reducing the number of bacterial fimbriae and affecting nucleic acid function. Nevertheless, these strategies face challenges related to bacterial resistance (Martin et al., 2019; Soleymani and Naghib, 2023).

Natural substances (e.g., aloe vera, chamomile, calendula, and propolis) for scaffold modification have been gaining interest because they mitigate the possibility of bacterial resistance (Kresnoadi et al., 2020). Propolis is a resin produced by bees to seal their hives, primarily during rainy seasons. Its anti-inflammatory, analgesic, wound-healing, and antimicrobial properties have been described previously (Altan et al., 2013; Przybyłek and Karpiński, 2019; Almuhayawi, 2020). These properties might allow for maintaining a balance between cells and bacteria in the early stages of bone grafting, preventing the effects of reactive oxygen species on bone cells exacerbated by surgery-induced injury and enabling cell proliferation that prevents bacterial apical migration (Hotta et al., 2020).

The present study aimed to evaluate the *in vitro* osteogenic behavior of Human Bone Marrow Stem Cells (bmMSCs) and assess the antimicrobial response to 3D-printed porous scaffolds.

## 2 Experimental procedure

### 2.1 Obtaining and characterizing the propolis extracts

Propolis was obtained from a community of beekeepers operating in the municipality of Tame, Colombia. All propolis samples were stored at 4°C before the extraction process. Ethanolic extracts of propolis (EEPs) were prepared by mixing 10 g of each propolis sample with 100 mL of 70% (v/v) ethanol. The mixture was then stirred for 24 h at a controlled speed of 120 rpm at room temperature and filtered by gravity. The filtrates were stored in the freezer for 12 h at −6°C and then re-filtered to remove waxes. The solvent was removed from the solutions using a rotary evaporator (RE-20000E, China) at 40°C and pressure of 2.5 bar until the volume was reduced to one-third of the initial volume. The antioxidant activity of the EEPs was assessed using the 2,2′-Azino-bis [3-ethylbenzothiazoline-6-sulfonic acid] (ABTS) assay and the 2,2-diphenyl-1-picrylhydrazyl (DPPH) free radical scavenging capacity assay.

The ABTS assay was performed using the method proposed by Re et al. (1999). The radical was generated through an oxidation reaction of ABTS with potassium persulfate. Then, 4 µL of the previously prepared EEPs were mixed with 196 µL of the ABTS solution in a phosphate buffer solution at pH 7.4. The resulting solutions were incubated at room temperature in the dark for 30 min, and absorbance was measured at 734 nm using a spectrophotometer (Zeiss, Germany). A solution of 50 μg/mL Trolox (6-hydroxy-2,5,7,8-tetramethylchroman-2-carboxylic acid) was used as the positive control. The results obtained were expressed as a percentage of antioxidant activity using the formula given in Eq. 1:

$$\%\;oxidant\;activity=\frac{\left(AABTS-AS\right)}{AABTS}x\;100$$ (1)

where AABTS denotes the absorbance of ABTS and AS denotes that of the sample. Values were expressed as the mean of three replicates. All comparisons were made using the ANOVA test with a Tukey *post hoc* test. Differences were considered significant for values of *p* ≤ 0.05.

The DPPH free radical scavenging capacity assay was performed using the method reported by Kumazawa et al. (2004) with some modifications. In a 96-well plate, 10 µL of EEPs and 190 µL of a methanolic DPPH solution were combined. The plate was shaken and then allowed to stand for 30 min at room temperature in the dark. Absorbance was measured at 517 nm using a spectrophotometer (Zeiss, Germany). A solution of 50 μg/mL Trolox was used as the positive control. The results were expressed as a percentage of antioxidant activity using the formula provided in Eq. 2:

$$\%\;oxidant\;activity=\frac{\left(ADPPH-AS\right)}{ADPPH}x\;100$$ (2)

where ADPPH denotes the absorbance of DPPH and AS denotes that of the sample. Values were expressed as the mean of three replicates. All comparisons were made using the ANOVA test with a Tukey *post hoc* test. Differences were considered significant for values of *p* ≤ 0.05.

### 2.2 Obtaining and characterizing the scaffolds

#### 2.2.1 Formulation and preparation of the ceramic paste

A fractional factorial design of experiments was used to obtain the optimized paste formulation for 3D printing of the scaffolds. The components of the mixture were considered as factors. As the response variable, the force required to extrude the suspension through a syringe was determined by applying force perpendicular to the plunger using a universal testing machine (Instron 3366 Universal Testing System) (MA, United States) with a 500N load cell, thus simulating the printing process. The components included NYADⓇ M1250 wollastonite powder (Paris, France) with an average particle size of 4 µm and acicular morphology, polyvinyl alcohol (PVA, Merck, Germany) as a binder; carboxymethyl cellulose (CMC, Sigma, United States) as a rheology modifier, and distilled water as the liquid fraction. The levels for each of these factors were selected as shown in Table 1.

**TABLE 1:** Factors used in the experimental design for paste formulation and their levels.

Component	Range of evaluation
Wollastonite	40%–60% w/v
PVA	3%–6% w/v
CMC	0%–0.3% w/w

The refinement of the formulation was carried out after establishing an initial formula and conducting a rheological fluidity analysis.

#### 2.2.2 Printing of the scaffolds

In the printing process, a DELTA WASP 2040 3D printer (Italy) was used with modifications to the print head, which allowed the attachment of a 5-mL syringe with an 18-G needle. The scaffolds were printed according to a gyroid infill pattern, i.e., a triply periodic minimal surface (TPMS). The printed samples had a cylindrical external shape of 15 mm in diameter and 5 mm in height. The G code for printing was developed using the Cura software (v. 4.13.1, Ultimaker, Netherlands).

For printing, a height of 0.4 mm was set for the first layer and 0.7 mm for the following layers using a line width of 0.3 mm. The infill density was 10% and the infill line distance was 1.5 mm. The TPMS gyroid geometry was used with an overlap of 5%. The flow rate was 20%, the print speed was 3 mm/s and the travel speed was 40 mm/s.

The printed samples were left to dry at room temperature for 24 h and then subjected to heat treatment in an oven (SX1700, Sentro Tech, United States). Briefly, the samples were further dried for 1 h at 200°C, then incubated at 500°C for 3 h to eliminate organic residues, and finally stored at 1180°C for 3 h, which corresponds to the sintering temperature of wollastonite particles. At the end of the process, the samples were left to cool naturally inside the oven.

#### 2.2.3 Impregnation of the scaffolds with propolis extracts

The scaffolds were desinfected with 70% ethanol for 30 min and impregnated with EEPs through vacuum absorption. Briefly, 20 μL of EEPs was placed on the scaffold’s surface and then subjected to a positive pressure in a chamber (Wiropress, BEGO, Germany) for 3 min. The absorption was verified by the color change of the scaffold and the process was repeated until the total volume absorbed was 150 μL.

#### 2.2.4 Evaluation of variations in pH

Bone regeneration devices might produce variations in the pH in the microenvironment, where they are implanted. Herein, the variations in pH observed when propolis-impregnated (pIS) and non-impregnated scaffolds (n-IS) were immersed in 100 mL of phosphate-buffered saline (PBS, Merck, Germany) was determined. The pH was measured using a PH700 benchtop pH meter (Apera, United States). Measurements were taken every hour for 70 h.

#### 2.2.5 Evaluation of antimicrobial activity

The antimicrobial activity of the propolis-impregnated and non-impregnated scaffolds was evaluated using a co-culture of *S. aureus* (ATCC 25175) and *S. epidermidis* (ATCC 12228) according to the procedure described by the Clinical and Laboratory Standards Institute (CLSI). The strains were pre-inoculated Mueller-Hinton agar (Merck, Germany) and incubated at 37°C for 24 h. To obtain the bacterial inoculate, the strains were grown to the exponential phase in BHI medium (Merck, Germany) at 37°C for 24 h and adjusted by diluting fresh cultures until a turbidity equivalent to 90 NTU (approximately 1.5 × 10^8^ CFU/mL) was obtained. Then, serial dilutions were made to a concentration of 1.5 × 10^6^ CFU/mL and mixed in equal parts to obtain the co-culture.

#### 2.2.6 Determination of the minimum inhibitory concentration (MIC)

To determine the MIC of *S. aureus and S. epidermidis* under monoculture conditions, serial dilutions were made in broth in 96-well microplates. Briefly, 50 μL of Mueller- Hinton broth (Merck, United States) and 50 μL of the EEP were added in each well, according to the procedure described by the CLSI with some modifications (Seidel et al., 2008). A stock solution of 50 mg/mL EEP was prepared and then diluted in Mueller-Hinton broth by serial dilutions (1:2, v/v) to achieve concentrations ranging from 50 to 1000 μg/mL in the microplates. To the wells containing different concentrations of EEP, 50 μL of bacterial suspension (approximately 1.5 × 10^6^ CFU/mL) was added. Then, 50 μL of resazurin solution (100 μg/mL) was added and the plates were incubated at 37°C for 2 h. Blue staining indicates lack of bacterial growth for *S. aureus* or *S. epidermidis*, whereas pink staining indicates bacterial growth. Ethanol in Mueller-Hinton broth (70% v/v) was used as a control for the solvent used in the extracts, and Mueller-Hinton broth was used as a negative control. MIC values were defined as the concentration that inhibited bacterial growth for both strains evaluated. The assay was performed in triplicate.

#### 2.2.7 ZOI

Suspensions of *S. aureus* and *S. epidermidis* (1.5 × 10^8^ CFU/mL) were prepared separately to assess the inhibitory effect using the Kirby–Bauer technique. Subsequently for each strain evaluated, 1 mL of suspension was taken and spread on plates containing Mueller-Hinton agar medium using a swab. The propolis-impregnated and non-impregnated scaffolds were placed onto inoculated plates. Paper discs of 5 mm diameter were soaked with saline solution (negative control) and chlorhexidine Digluconate at 0.2% (positive control, Farpag, Colombia). Finally, the plates were incubated at 37°C for 24 h and its antimicrobial activity was evaluated by measuring the diameter of the growth inhibition zone in millimeters (including the disc diameter) using ImageJ software.

#### 2.2.8 Co-culture biofilm formation assay

Propolis-impregnated and non-impregnated scaffolds were used for the co-culture biofilm formation assay. Each group of scaffolds was incubated with 1 mL of the co-culture for 24h and 48 h at 37°C. The culture medium was renewed every 16 h. After incubation, the scaffolds were washed three times with PBS to remove the non-adhered bacteria. Viability tests were conducted on the biofilm adhered to the discs. To complete this, 3-(4,5-dimethylthiazol-2-yl)-2,5-diphenyltetrazolium bromide (MTT) was added and incubated for 2 h. Dimethyl sulfoxide was then added, and absorbance was measured at 550 nm using a spectrophotometer (Zeiss, Germany).

#### 2.2.9 Cytotoxicity and biocompatibility assays

A culture of human bone marrow derived MSCs (bmMSCs) was used for cytotoxicity and biocompatibility assays. The bmMSCs were kindly provided by Dr. Yvonne Roger from the Orthopedic University Clinic of the Hannover Medical School. The bmMSCs (6th–8th passages) were seeding on propolis-impregnated and non-impregnated scaffolds (premoistened in supplemented medium overnight) at a density of 25,000 cells/cm^2^. As a control, cells were seeding in a 24-well plate (TPP, Switzerland). Non-impregnated (n-IS) and scaffolds impregnated (IS) with 150 µL of propolis were evaluated; they were sterilized and then immersed in PBS (Merck, Germany) for 72 h to avoid the initial release of calcium ions that could damage the cells. The scaffolds were cultured in 24-well plates (TPP, Switzerland) in growth medium supplemented with Dulbecco’s modified Eagle medium (Bio&Sell, Germany) containing 2.3% (v/v) stable glutamine, 2.3% (v/v) HEPES, 0.002% (v/v) FGF-2, 11.6% (v/v) fetal bovine serum (Bio&Sell, Germany), and 1.2% (v/v) penicillin/streptomycin. The medium was replaced every 2 days during the first 7 days of culture (Leal-Marin et al., 2021). Subsequently, the cells were cultivated to a differentiation medium by adding 5 mM β-glycerophosphate (Merck, Germany), 50 mM ascorbic acid (Merck, Germany), and 10 nM dexamethasone (Merck, Germany) for 28 days (Pelaez-Vargas et al., 2012).

#### 2.2.10 Cell proliferation assay and mineralization

Cell proliferation was evaluated at day (1, 3, 7, 14, 21 and 28) using Alamar Blue assay. Cultures were incubated for 1.5 h and fluorescence was quantified using a multiplate reader (Tecan Infinite M200 Nanoquant, United States) at 570 nm (excitation wavelength) and 600 nm (emission wavelength) (Leal-Marin et al., 2021). The results were expressed as the percentage of viability found in the scaffolds relative to the control samples sown in the wells without scaffolds. The assays were conducted in triplicate. Additionally, cell morphology and adhesion on scaffolds were characterized via SEM (S3400N, Hitachi, Japan). Cells were treated in cacodylate buffer (0.1M, Merck, Germany), fixed in 3% glutaraldehyde (Merck, Germany) and dehydrated using a series of graded ethanol solutions (25%–99%, v/v). After drying overnight, the samples were coated with a 35 nm Au-Pd layer (Minisputter–SC7620, Quorum Technologies, United Kingdom).

A histochemical characterization was conducted for a qualitative assessment of osteogenesis, which involved identifying calcium deposits on the scaffolds using von Kossa staining, and Alizarin red staining, as well as, measuring alkaline phosphatase (ALP) activity. A total of 25,000 cells/cm^2^ were seeded on the propolis-impregnated and non-impregnated scaffolds in 24-well plates. These cells were then maintained under the culture conditions described above and cultured in a differentiation medium from day 7 to day 28. For staining, measurements were taken on days 21 and 28, during which the cells were fixed with 3% glutaraldehyde (Merck, Germany) for 15 min and subsequently washed with PBS (Merck, Germany).

For von Kossa staining, 1 mL of 1% silver nitrate (Merck, Germany) was added to the scaffolds, which were then exposed to UV light for 1 h. Subsequently, the scaffolds were rinsed with distilled water and 1 mL of 5% sodium thiosulfate (Merck, Germany) was added for 3 min. Finally, the scaffolds were rinsed again with distilled water and allowed to air dry (Sundaram and Milton, 2017).

For Alizarin red staining, scaffolds were treated with 4 nM sterile Alizarin red solution (Merck, Germany) for 2 min and washed with deionized water. Then, 10 mM HCl in 70% ethanol was added for 15 s. The scaffolds were left to air dry (Abbasi et al., 2020).

For ALP staining, a solution of Na-naphthyl phosphate (Merck, Germany) with Tris buffer (Sigma, United States) was used. The solution was stirred for 10 min, added with fast blue (Sigma, United States), and then stirred for 5 min. This solution (1 mL) was added to the scaffolds, following which the scaffolds were stored in the dark for 1 h, washed with PBS, and then allowed to air dry.

The scaffolds subjected to these stains were observed under an optical stereomicroscope (Stemi DV4, Carl Zeiss, Germany). Scaffolds without cell culture with each of the stains were used as a staining control on the material. These experiments were conducted in triplicate.

#### 2.2.11 Mechanical properties

The scaffolds were subjected to quasi-static monotonic compression tests using a UTS (Universal Testing System, Instron 3366, Instron, MA, United States) with a 10 kN load cell at a rate of 0.5 mm/min. The tests recorded compression load and displacements, with the maximum load or failure load used to determine the maximum compressive strength of each scaffold sample. Samples were tested after sintering and following 21 and 28 days of cell culture to identify any potential mechanical response changes.

### 2.3 Statistical analysis

All experiments were performed in triplicate. The results were expressed as mean ± standard deviation. Statistical analysis was performed using the SPSS Statistics software (V21, IBM, United States). An unpaired Student’s t-test was used to assess significance of differences between experimental groups and comparison of % viability between different days was performed using the one-way ANOVA test with *post hoc* Tukey method. Values of *p* ≤ 0.05 were considered statistically significant.

## 3 Results

### 3.1 Antioxidant activity of propolis extracts

**FIGURE 1:**
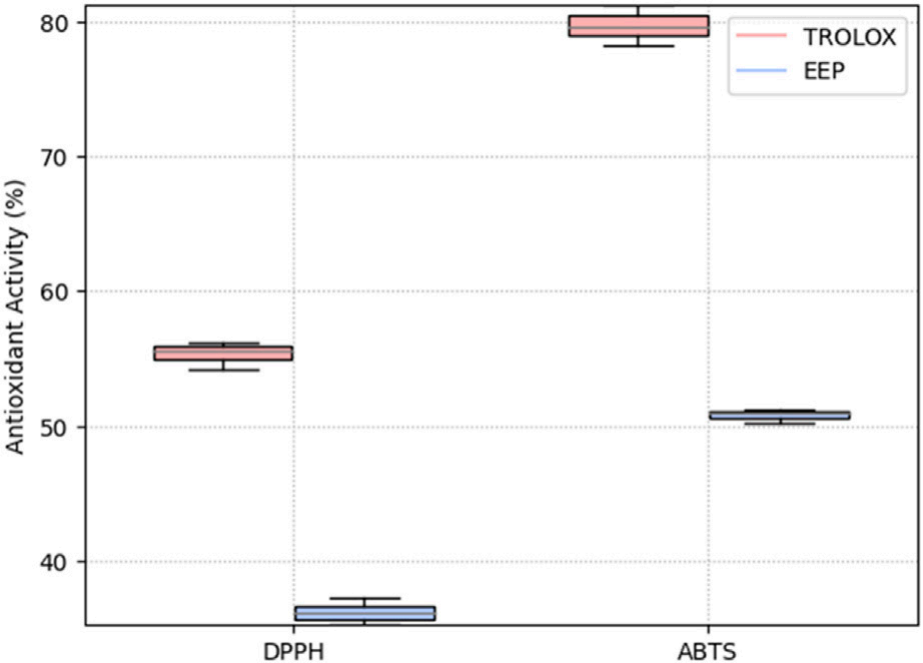
Antioxidant activity of ethanolic extracts of propolis (EEPs) and positive control Trolox solution (Trolox) determined via DPPH and ABTS assays.

The tests conducted to assess the antioxidant activity of the EEPs demonstrated their effectiveness against the two analyzed radicals. The DPPH method identifies low-polarity compounds capable of neutralizing DPPH free radicals through hydrogen donation, while the ABTS method assesses the activity of both hydrophilic and lipophilic compounds. The results of the antioxidant activity of the EEPs are presented in Figure 1. The EEPs exhibited an antioxidant activity of 55.4% ± 1% with the DPPH method and 36.2% ± 1% with the ABTS method. Additionally, the Trolox solution, used as a control, displayed values of 79.7% ± 2% and 50.8% ± 1% in the DPPH and ABTS assays, respectively. These results are promising, indicating that the EEPs contain both polar and hydrophilic components, enabling them to effectively neutralize free radicals through diverse mechanisms.

### 3.2 Ceramic paste formulation

**FIGURE 2:**
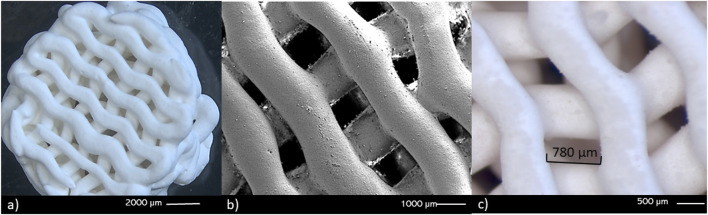
Stereomicroscopy **(A, C)** and SEM **(B)** images of the scaffolds obtained through 3D printing. **(A, B)** the curvature in the printed layers can be observed, as well as the **(C)** 3D structure of the macropore walls.

From the fractional factorial design of experiments, a ceramic paste formulation was obtained with the following composition: 59.97% (w/w) wollastonite, 0.03% (w/w) CMC, 37.75% (w/w) deionized water, and 2.25% (w/w) PVA aqueous solution. The ceramic paste exhibited non-Newtonian fluid rheological behavior, which was fitted to the Herschel–Bulkley model, allowing for the 3D printing of cylindrical scaffolds. These scaffolds had a radius of 5 mm, height of 5 mm, layers of 500 μm, and pore size of 780 µm. Figure 2 shows the SEM images of the printed scaffolds, showing the curvature achieved in each of the printing layers and the size and shape of the pores obtained using the TPMS.

### 3.3 Variations in pH

**FIGURE 3:**
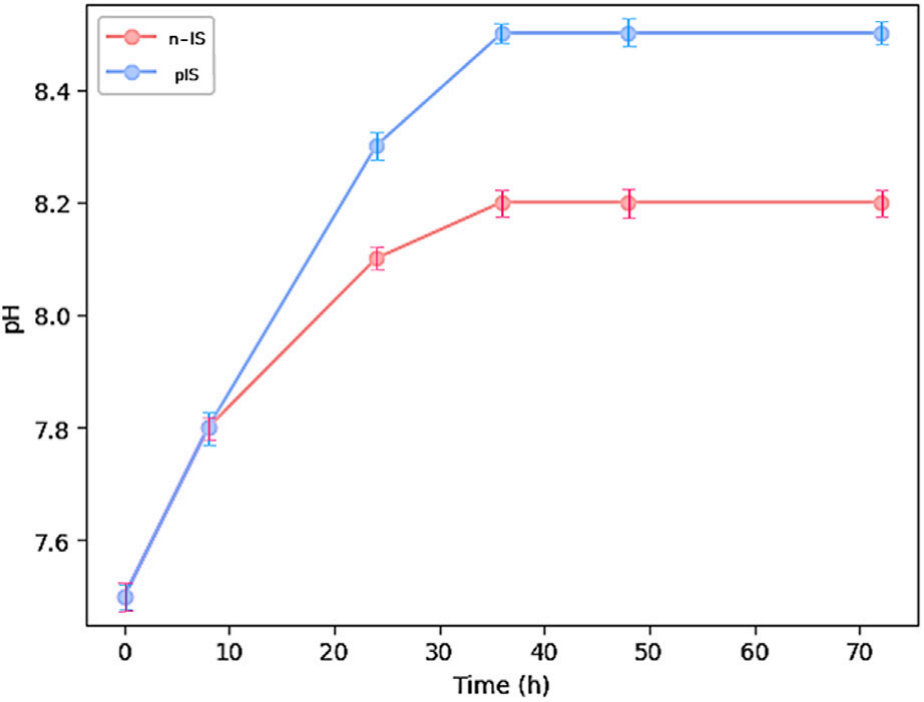
pH measurement of propolis-impregnated scaffolds (IS) and non-impregnated scaffolds (n-IS) over 72 h.

Figure 3 displays the curves representing variations in pH over time for both the propolis-impregnated and non-impregnated scaffolds. At the start of the test, the distilled water in which the samples were immersed had a pH value of 7.5. During the initial 8 h, this value remained unchanged, regardless of the type of sample being immersed. However, after 12 h, an increase in pH was observed in the solution containing the propolis-impregnated scaffolds, reaching a pH value of 8.5 after 30 h and remaining stable thereafter. Conversely, the solutions with the non-impregnated reached a maximum pH of 8.2. These variations suggests that the EEPs create more alkaline environments, which may have a detrimental effect on acidophilic bacterial species.

### 3.4 Antimicrobial activity

Antimicrobial activity was evaluated using three different methodologies: MIC and inhibition zones were studied from single strain cultures, and biofilm formation was evaluated on the scaffolds under co-culture conditions. The MIC for each strain evaluated were defined as the lowest concentration at which bacterial growth inhibition was observed. The EEPs exhibited inhibitory effects against the two strains evaluated, with a propolis concentration of 1.1 ± 0.5 mg/mL required to inhibit *S. aureus* and 1.7 ± 0.3 mg/mL required to inhibit *S. epidermidis*.

**FIGURE 4:**
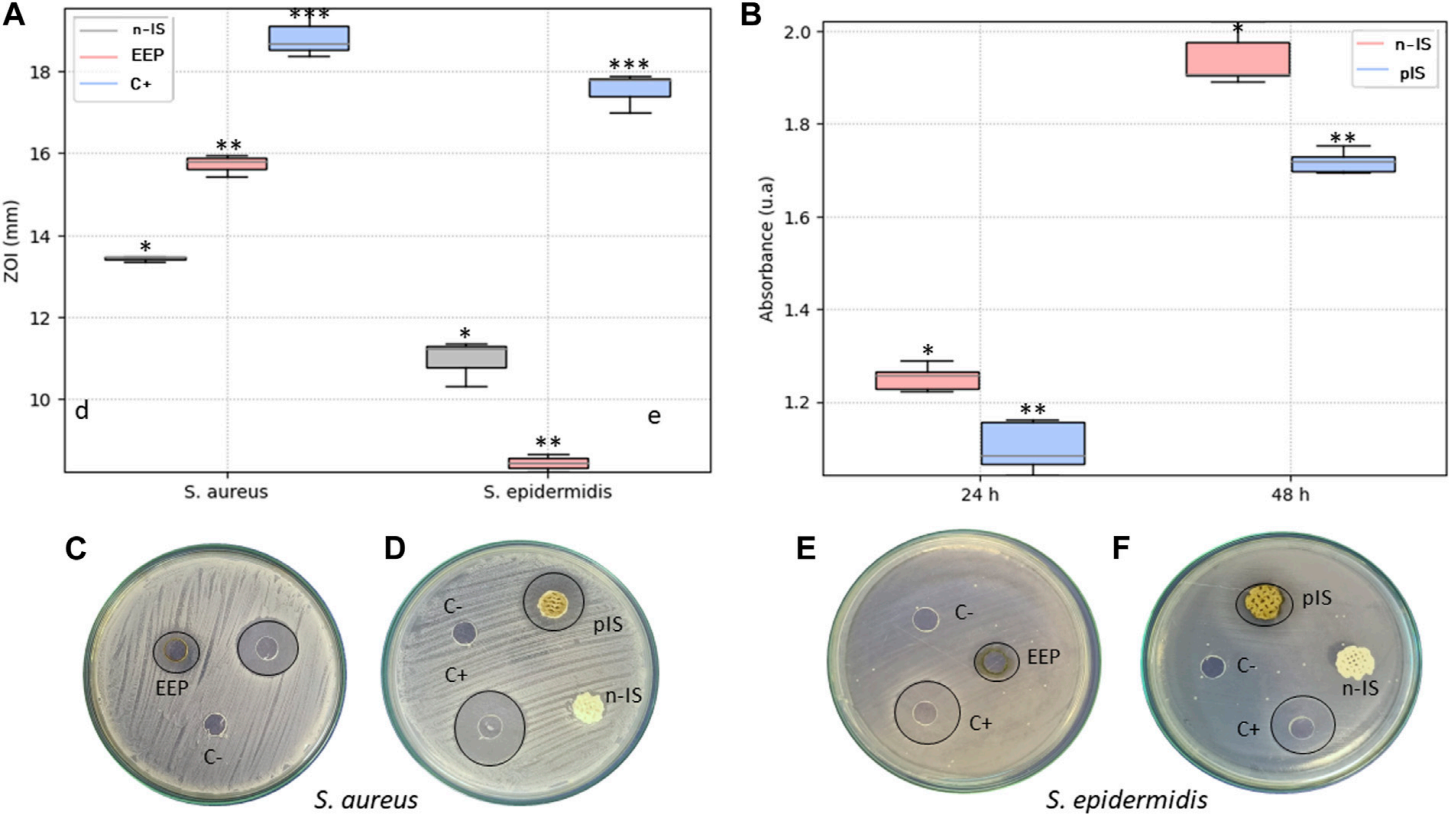
Antimicrobial activity. **(A)** Measurement of the inhibition zones of the EEPs, untreated scaffolds (n-IS), and treated scaffolds (pIS) against *S. aureus* and *S. epidermidis*. **(B)** Viability of biofilm formation after 24 and 48 h of culture on n-IS and pIS against a co-culture of *S. aureus* and *S. epidermidis*. **(C, D)** Images of the inhibition zones of the EEPs, chlorhexidine digluconate (C+), and saline solution (C−) against *S. aureus* and *S. epidermidis.* **(E, F)** Measurement of the inhibition zones of the US and TS against *S. aureus* and *S. epidermidis*. Results are expressed as means ± SD (*n* = 3). Significance levels are denoted as follows: **p ≤ 0.05 indicates a significant difference, while (ns) denotes a non-significant difference. All the measurements of the inhibition zones are expressed in mm.

Figure 4 displays the measurements of the inhibition zones observed during the test. These measurements indicate the antimicrobial effect of the EEPs against the two strains, with a stronger activity against *S. aureus* compared with *S. epidermidis*. These findings align with the results of the MIC test, where the lower concentration of propolis required to inhibit the growth of *S. aureus* suggests greater sensitivity to it, resulting in larger inhibition zones compared with those observed for *S. epidermidis* at the same propolis concentration. However, notably, the antimicrobial activity of propolis was significantly lower than that of the positive control against both strains. The positive control exhibited inhibition zones of 18.87 ± 0.6 mm and 17.54 ± 0.5 mm against *S. aureus* and *S. epidermidis*, respectively, whereas the EEPs exhibited zones of 15.72 ± 0.3 mm and 8.45 ± 0.2 mm, respectively, and the pIS exhibited zones of 13.43 ± 0.07 mm and 10.97 ± 0.6 mm, respectively.

In Figure 4, the results of biofilm formation by the co-culture reveal a significant reduction in bacterial viability on the pIS compared with the n-IS at both evaluated time points. After 24 h of culture, the absorbance value in the pIS scaffolds was 1.10 ± 0.05 u. a., whereas in the n-IS, it was 1.25 ± 0.03 u.a. After 48 h of culture, the pIS scaffolds exhibited a value of 1.71 ± 0.06 u.a., whereas the n-IS exhibited a value of 1.94 ± 0.1 u. a., clearly demonstrating the effective control of bacterial viability provided by the EEPs.

### 3.5 Cell proliferation and biocompatibility

The bmMSCs proliferation was assessed using the Alamar Blue assay with different time-points (1–28 days). The % viability values for the pIS group exhibited statistically significant differences (*p =* 0.01) between early stages (1 and 7 days) and later stages (14, 21, and 28 days). The n-IS group showed a similar trend (*p =* 0.01). This behavior indicates the transition from the proliferation to the differentiation phases of cultures.

**FIGURE 5:**
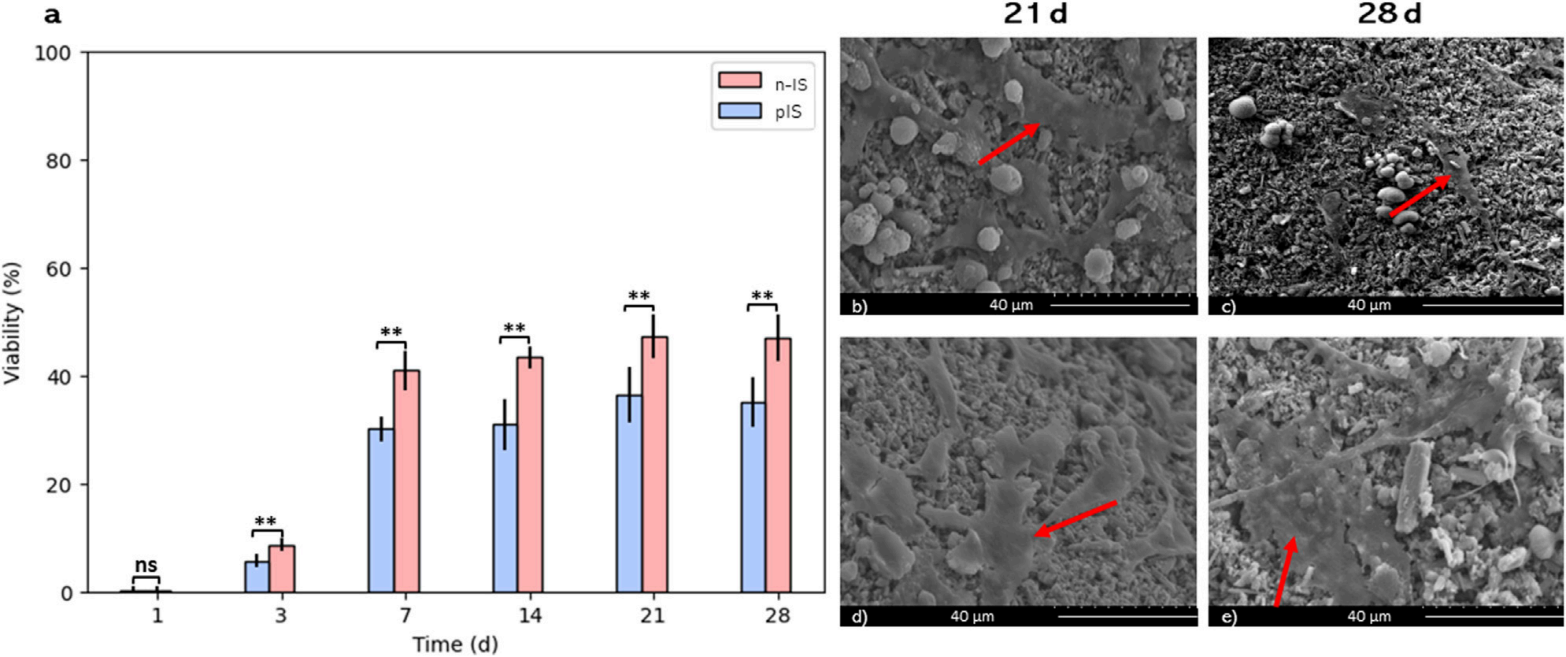
bmMSCs cell proliferation assays. **(A)** Viability (%) propolis-impregnated scaffolds (pIS) and non-impregnated scaffolds (n-IS). SEM images of bmMSCs adhesion on **(B, C)** n-IS and **(D, E)** pIS after 21 and 28 days of culture. Results are expressed as means ± SD (*n* = 3). Symbol (**) denote statistically significant differences (*p* ≤ 0.05). Arrows indicate cells at different spreading stages.

A viability of 50% for the cell culture on the non-impregnated scaffolds was reached by day 21 and maintained until day 28 of culture. On the propolis-impregnated scaffolds, a viability close to 40% was achieved and maintained through day 28. Figure 5 shows SEM images highlighting the cell morphology (arrows) found for both scaffold groups, consistent with the morphology of stem cells reported by other authors (Gutiérrez-Prieto et al., 2019; Qiu et al., 2019; Bahraminasab et al., 2021).

**FIGURE 6:**
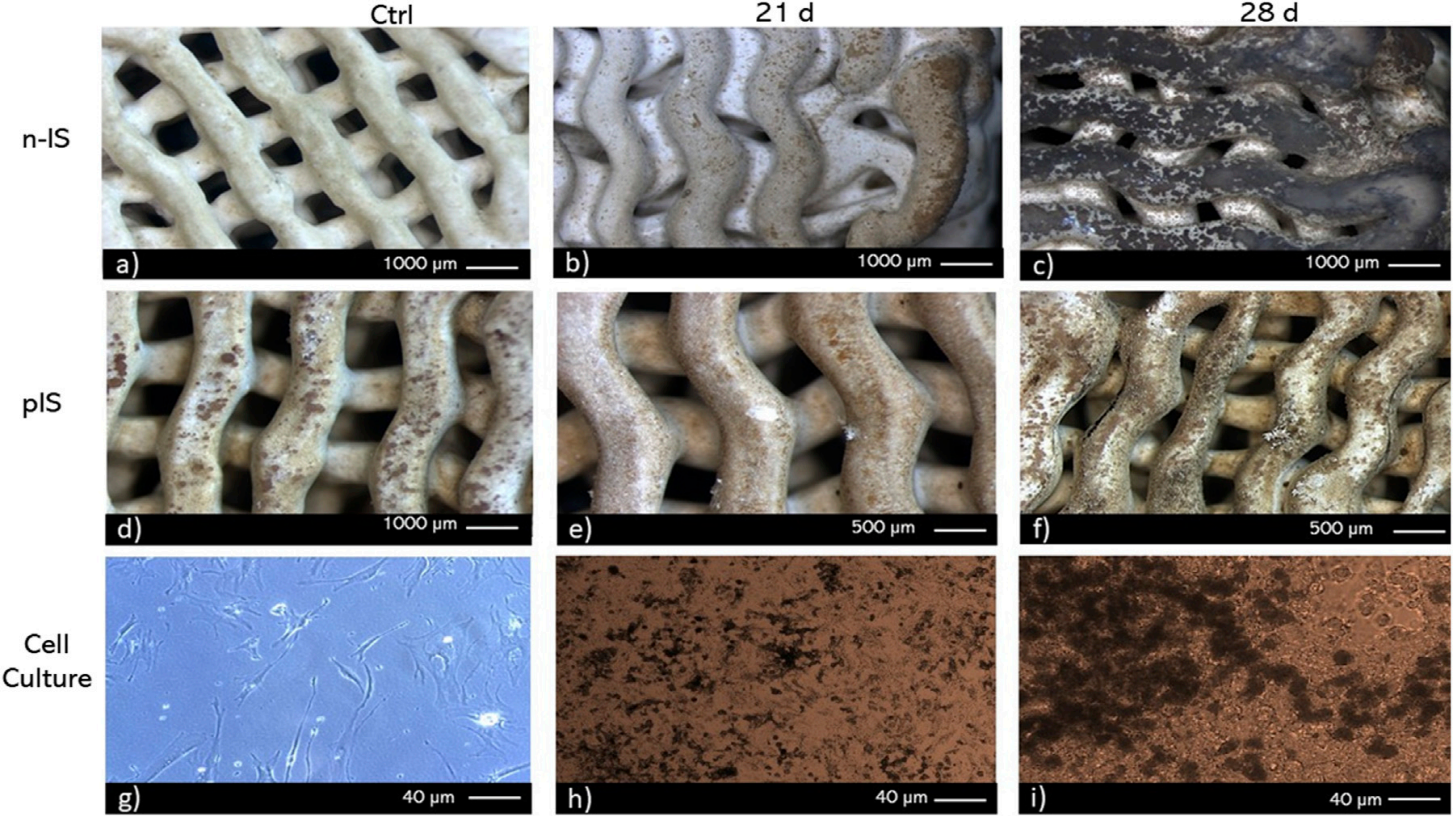
von Kossa staining of bmMSC cultures on **(A–C)** non-impregnated scaffolds (n-IS), **(D–F)** propolis-impregnated scaffolds (pIS), and **(G–I)** tissue culture polystyrene TCPS. Time points: 21 and 28 days. Control without cells (Ctrl).

**FIGURE 7:**
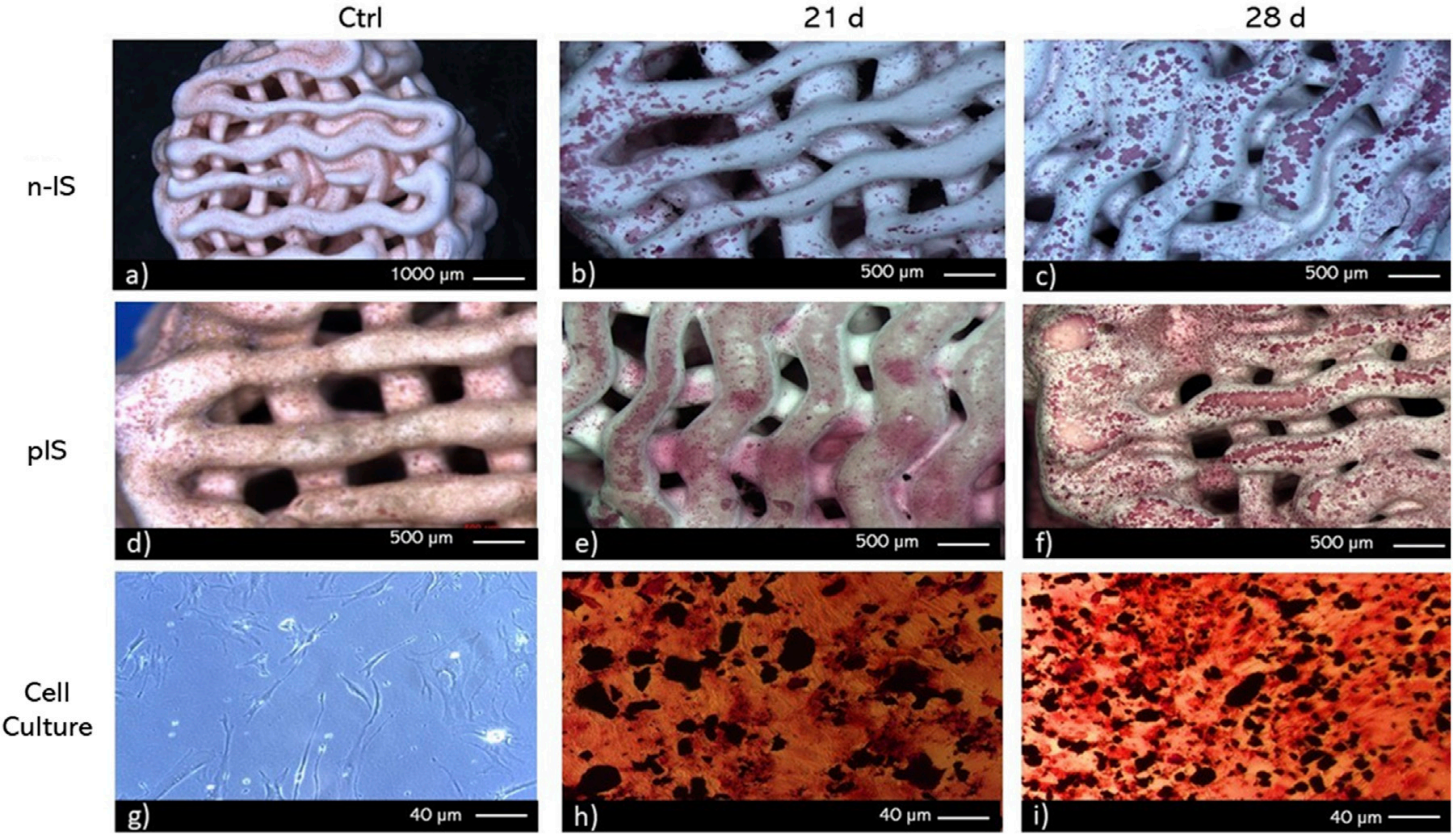
Alizarin red staining of bmMSC cultures on **(A–C)** non-impregnated scaffolds (n-IS), **(D–F)** propolis-impregnated scaffolds (pIS), and **(G–I)** TCPS. Time points. 21 and 28 days. Control (Ctrl).

To identify scaffold regions with calcium deposits and ALP activity, histochemical techniques were employed. To visualize calcium deposits two methods were used von Kossa staining (Figure 6) and Alizarin red staining (Figure 7). After 28 days of culture, von Kossa staining revealed black staining indicative of calcium deposits on 70% of the top layer of the n-IS. Conversely, the pIS exhibited brown staining, which intensified over time (Figures 6E, F). Scaffolds used as staining controls did not display any significant color change upon the application of staining reagents.

Contrary to the findings obtained with von Kossa staining, calcium deposits were more prevalent in the pIS than in the n-IS after 28 days of culture. Notably, with this staining, some color change was also observed in the control scaffolds (those not subjected to cell culture), although at a lower intensity. To prevent false positives, only regions displaying intense staining were considered positive results. Alizarin red staining revealed the presence of calcium deposits in the internal layers with pronounced staining. This pattern was consistent at both time points and in both scaffold groups.

**FIGURE 8:**
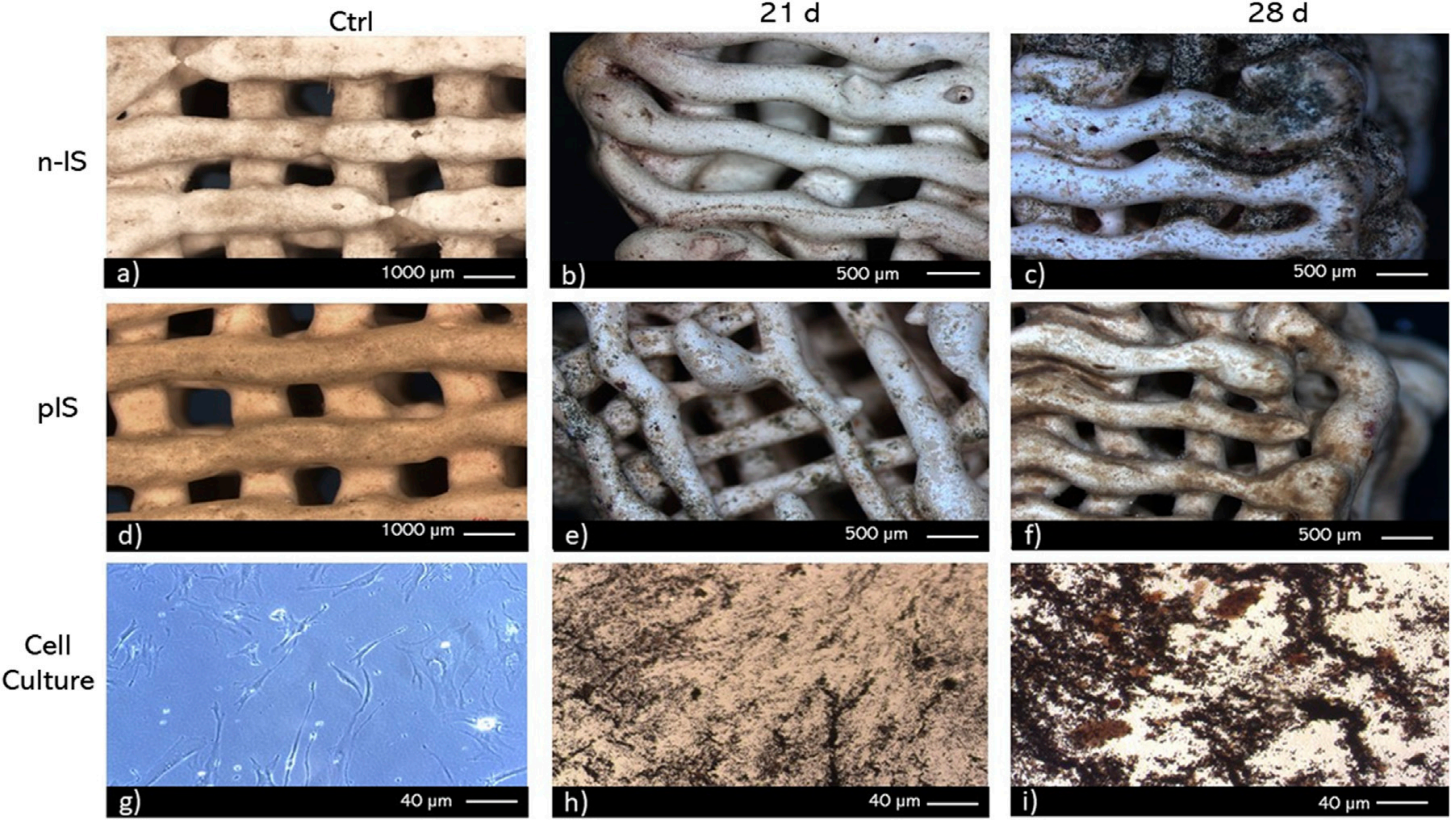
ALP staining of bmMSC cultures on **(A–C)** untreated scaffolds (n-IS), **(D–F)** propolis-impregnated scaffolds (pIS), and **(G–I)** TCPS. Time points. 21 and 28 days. Control (Ctrl).

Additionally, staining was performed to visualize ALP activity, as it serves as a marker for the initial phase of osteogenic differentiation. The results of the ALP staining are presented in Figure 8. After 28 days of culture, ALP activity exhibited higher intensity in the n-IS. However, ALP activity was also detected in the pIS after 21 days of culture. The regions displaying the highest activity were primarily located at the corners and areas with pronounced curvatures. In comparison, the scaffolds used as staining controls exhibited a faint brown hue, significantly less intense than that observed in the scaffolds exposed to cell culture.

### 3.6 Mechanical test

**FIGURE 9:**
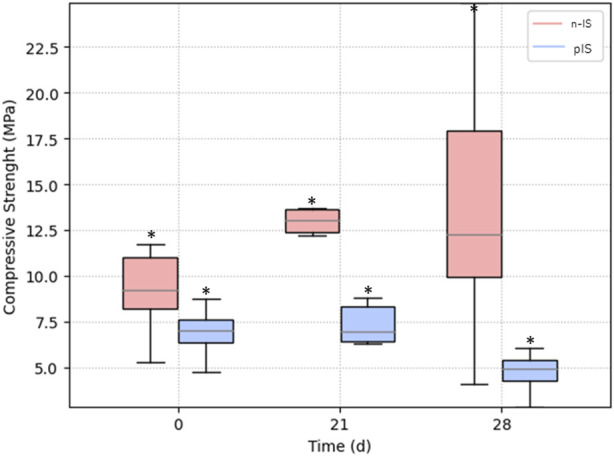
Mechanical response of propolis-impregnated scaffolds (pIS) and non-impregnated scaffolds (n-IS). No significant differences were observed between sample sets.

The compressive strength of the scaffolds was measured before and after cell proliferation tests for 21 and 28 days (Figure 9). Statistical analysis indicated no significant differences between the groups. Additionally, the compressive strength of the scaffolds remained within the typical range (0.1–16 MPa) for trabecular bone (Rincón-Kohli and Zysset, 2009; Gerhardt and Boccaccini, 2010; Kaur et al., 2019). These findings suggest that the propolis impregnation treatment does not significantly impact the mechanical response or function of the scaffolds. Notably, although there were no significant differences in the mechanical results, an evident increase in variability in the scaffolds’ results was observed after 28 days of cell proliferation compared with the results obtained at 0 and 21 days (Figure 9). This change can be attributed to cell adhesion to the scaffold, resulting in the removal of wollastonite particles from certain areas of the surface, thus affecting the repeatability of the results.

## 4 Discussion

The selected design of experiments has been widely employed for formulating and optimizing ceramic pastes (Yang and Van Olmen, 2004; Yoon and Lee, 2004; Renteria et al., 2019). It allows for a specific combination of parameters, particularly crucial in 3D printing of ceramics, where obtaining pastes with precise rheological properties is essential for layer-by-layer design. This is especially challenging when developing devices with complex geometries, such as ceramic scaffolds with TPMS gyroid geometries. In this study, the choice of TPMS gyroid geometries was deliberate because research has shown that they promote cell proliferation owing to their sinusoidal structures, creating interconnected pores that facilitate oxygen diffusion and nutrient distribution. These structures also optimize cellular distribution, reducing the stress effects triggered by environmental conditions that can lead to replicative senescence (Abueidda et al., 2019). TPMS geometries have demonstrated mechanical properties suitable for bone tissue engineering applications, often comparable to other TPMS structures widely used in this field (Abueidda et al., 2019; Castro et al., 2019). The mechanical compressive strength evaluation of the scaffolds yielded values within the same range as trabecular bone (0.1–16 MPa) (Rincón-Kohli and Zysset, 2009; Gerhardt and Boccaccini, 2010; Kaur et al., 2019). This supports the idea that the porosity achieved through additive manufacturing of ceramic scaffolds is well-suited for repairing bone lesions. Furthermore, the impregnation of the scaffolds with propolis had no detrimental effects on their mechanical properties.

The propolis extracts exhibited antioxidant activity using two methodologies. However, the antioxidant activity of the propolis-impregnated scaffolds was notably lower than that of the control (Trolox Solution). Our activity values align with other studies, which have reported antioxidant activity against the DPPH free radical ranging from 17% to 70% and against the ABTS free radical ranging from 5% to 28% (Ahn et al., 2007). This antioxidant activity can be attributed to the presence of specific components such as phenols and flavonoids found in propolis from this region (Moreno et al., 2023). Furthermore, the antioxidant activity, along with the presence of phenolic compounds and flavonoids, may be associated with antimicrobial activity. The evaluated EEPs demonstrated antimicrobial activity against the two strains used as well as their co-culture. Propolis is known to employ multiple mechanisms to inhibit bacterial growth, including damaging the cell membrane, altering membrane potential and ATP production (Przybyłek and Karpiński, 2019), inducing oxidative stress, and inhibiting bacterial enzymatic activity (Mirzoeva et al., 1997). Notably, propolis is typically more effective against gram-positive bacteria than against gram-negative ones and tends to function better against aerobic bacteria than against anaerobic ones. The two strains evaluated in this study are gram-positive and facultative anaerobes. They also exhibit resistance to methicillin (Eladli et al., 2019) and possess a high virulence factor, allowing them to form biofilms easily (Periasamy et al., 2012). Additionally, these two bacteria can form co-cultures, increasing their capacity to create biofilms, thus making them more virulent. The EEPs were demonstrated to be effective against the co-culture. This significant reduction in efficacy may be attributed, in part, to the pH conditions in the pIS, which create a more basic microenvironment than that in the n-IS. These pH conditions are detrimental to the bacterial strains, as they typically thrive in acidic or neutral pH conditions (Iyer et al., 2021).

In terms of biocompatibility and proliferation, the propolis-impregnated scaffolds exhibited statistically non-significantly lower cell viability and proliferation than the non-impregnated scaffolds. This effect can also be attributed to the pH conditions in the microenviroment. Although a pretreatment was applied to neutralize this effect, the pH values of the pIS scaffolds were slightly higher than that of the n-IS. Propolis often contains resinous components (Huang et al., 2014) that may hinder the cell proliferation process, although they do not completely inhibit it. In previous studies, propolis has been used in bone tissue repair processes, demonstrating an increase in bmMSC cell proliferation at concentrations of <400 g/mL (Elkhenany et al., 2019). However, very high concentrations can lead to decreased proliferation, with cytotoxic effects typically observed at concentrations of >2000 g/mL (Bufalo et al., 2009; Elkhenany et al., 2019). Additionally, the presence of propolis has been shown to enhance the proliferation capacity of periodontal ligament fibroblasts and stem cells derived from exfoliated primary teeth (Ahn et al., 2007; Chew Shi Fung et al., 2015). Therefore, studies such as the present one, using different concentrations of propolis to assess its antimicrobial activity and cellular response, are of interest. The incorporation of propolis into wollastonite scaffolds has been demonstrated to increase proliferation percentages and, consequently, the mineralization process. Stainings used to visualize metabolic activity indicated that pIS do not impede osteogenesis processes, as evidenced by the presence of calcium deposits and ALP activity. Although the black staining observed in the von Kossa staining, interpreted as positive, was lower on the pIS than on the n-IS, sizable regions still showed a positive result. Wollastonite is well known for its biocompatibility in the field of bone tissues. The results obtained in this study are consistent with those reported by others in this regard (Porter et al., 2009; Jahan and Tabrizian, 2016; Carvalho et al., 2021; Zenebe, 2022). Finally, it has been well reported that uncorrected microenvironment acidosis can lead to a reduction of alkaline minerals in bone by osteoblasts, and there is an increase in osteoclast resorptive activity (Brandao-Burch et al., 2005). Our pH measurements showed values close to 8.4 for pIS and 8.2 for n-IS achieved at 40 h. These results are consistent with reported findings, suggesting that within the range up to pH 8.4, any increase above pH 7.2 is beneficial for proliferation and mineralization (Galow et al., 2017).

The porous scaffolds created through 3D printing with TPMS gyroid geometry exhibited mechanical properties suitable for repairing trabecular bone tissue. Additionally, impregnating the scaffolds with EEPs demonstrated effective control over bacterial growth associated with the development of osteomyelitis, a common cause of implant failure in bone repair procedures. Specifically, antimicrobial activity was observed against *S. aureus* and *S. epidermidis*. This antimicrobial effect may be attributed to the presence of phenolic compounds and flavonoids, which are also responsible for the antioxidant activity identified in this study. Moreover, the scaffolds impregnated with EEPs promoted the proliferation of stem cells derived from bone marrow, along with progress in the mineralization process after 28 days of culture. These findings open new avenues for further research on utilizing EEPs to maintain antimicrobial activity while minimizing their impact on the cellular microenvironment.

## Data Availability

The raw data supporting the conclusion of this article will be made available by the authors, without undue reservation.
